# An uncommon cause of tinea: Trichophyton violaceum in a German kindergarten – outbreak report and quantitative analysis of epidemiological data from Europe

**DOI:** 10.3205/dgkh000405

**Published:** 2022-01-27

**Authors:** Claudia Feußner, Sigrid Karrer, Benedikt M. J. Lampl

**Affiliations:** 1 Regensburg Department of Public Health, Regensburg, Germany; 2 Department of Dermatology, University Medical Center Regensburg, Regensburg, Germany; 3 Department of Epidemiology and Preventive Medicine, Faculty of Medicine, University of Regensburg, Regensburg, Germany

**Keywords:** dermatomycosis, kindergarten, Trichophyton violaceum, outbreak, epidemiology

## Abstract

**Background**: Global mobility is increasingly associated with the emergence of “unusual” infectious agents. At the beginning of 2019, a putative outbreak of *Impetigo contagiosa* occurred in a kindergarten in Regensburg, Germany, that was mainly attended by children with a migrant background. After thorough examination, the outbreak was classified as infection with *Trichophyton (T.)* violaceum.

**Methods**: Based on case investigations, infection control measures, disinfection, and cleaning were implemented. Microscopy of native specimens, fungal cultures, and polymerase chain reaction were used for diagnosis. Additionally, a systematic literature search in Medline, followed by a quantitative analysis of epidemiological data from Europe, were performed.

**Results**: Between January and November 2019, 12 cases of tinea were diagnosed in 7 educators and 2 household members. Children were initially not affected. *T. violaceum* was only detected in 2 patients. No extensive screening measures were carried out after risk-benefit assessment. Studies on *T. violaceum* in Europe are heterogeneous, and the number of cases and the prevalence vary considerably. The pathogen is mainly found in children of African descent who clinically present with *tinea capitis*.

**Discussion**: In the present case, the source of infection and the chain of transmission remained unclear. The pathogen could only be diagnosed in 2 cases. In Europe, the (re)emergence of pathogens such as *T. violaceum* is likely to be caused by increasing migration and travel. Pathogens should be identified for epidemiological reasons in all cases. In outbreaks, measures must be adapted to the dynamics of the individual outbreak after assessment of the risks, benefits, and proportionality.

## Zusammenfassung

**Hintergrund**: Globale Mobilität wird zunehmend mit dem Aufkommen „ungewöhnlicher“ Infektionserreger in Verbindung gebracht. Anfang 2019 kam es in einem Kindergarten in Regensburg, Deutschland, der hauptsächlich von Kindern mit Migrationshintergrund besucht wurde, zu einem mutmaßlichen Ausbruch von *Impetigo contagiosa*. Nach eingehender Untersuchung wurde der Ausbruch als Infektion mit *Trichophyton (T.)violaceum* eingestuft.

**Methoden**: Auf der Basis von Falluntersuchungen wurden Infektionsschutz-, Desinfektions- und Reinigungsmaßnahmen implementiert. Zur Diagnostik kamen Mikroskopie von Nativpräparaten, Pilzkulturen und Polymerase-Kettenreaktion zum Einsatz. Ergänzend wurde eine systematische Literaturrecherche in Medline mit anschließender quantitativer Analyse epidemiologischer Daten aus Europa durchgeführt.

**Ergebnisse**: Zwischen Januar und November 2019 wurden 12 Fälle von Tinea bei 7 Erziehern und 2 Haushaltsmitgliedern diagnostiziert. Kinder waren zunächst nicht betroffen. *T. violaceum* wurde nur bei 2 der Patienten nachgewiesen. Nach Nutzen-Risiko-Abwägung wurden keine umfangreichen Screening-Maßnahmen durchgeführt. Studien zu *T. violaceum* in Europa sind heterogen, die Fallzahlen und die Prävalenz variieren erheblich. Der Erreger wird hauptsächlich bei Kindern afrikanischer Abstammung gefunden, die sich klinisch mit einer *Tinea capitis* präsentieren.

**Diskussion**: Im vorliegenden Fall blieben die Infektionsquelle und die Übertragungskette unklar. Der Erreger konnte nur in 2 Fällen diagnostiziert werden. In Europa wird das (Wieder-)Auftreten von Krankheitserregern wie *T. violaceum* wahrscheinlich durch zunehmende Migration und Reisen verursacht. Krankheitserreger sollten grundsätzlich aus epidemiologischen Gründen bei allen Fällen identifiziert werden. Bei Ausbrüchen müssen die Maßnahmen nach Abwägung von Risiken, Nutzen und Verhältnismäßigkeit an die Dynamik des einzelnen Ausbruchs angepasst werden.

## Background

*Trichophyton (T.) violaceum* is a pathogen of the *Trichophyton rubrum* complex, which is rather rare in Europe, but currently endemic mainly in Africa and Asia. *T. violaceum* usually causes tinea capitis in children from endemic areas. *T. violaceum* spreads along the hair follicles within the hair shafts (endothrix infection) and may cause scaling plaques on the skin. These plaques are often associated with minimal inflammatory reactions but may be protracted and become chronic. Dermatophytes grow slowly at temperatures under 30°C and do not result in systemic fungal infection [1]. The anthropophilic *T. violaceum* dermatophyte is transmitted from human to human. Transmission via objects is also possible. Therapy usually consists of the topical application of imidazole or cyclopyroxolamine [1], [2], [3], [4].

The outbreaks of *Microsporum audouinii* in a daycare center in Munich in 2011 and in daycare centers and schools in Bonn in 2015 showed that species that are endemic in Africa and Asia but not autochthonous in Europe may become relevant for infection control because of increased migration and travel [5], [6], [7], [8]. 

*Impetigo contagiosa*, in contrast, is a superficial skin infection caused by *Staphylococcus* *aureus* or streptococci, which are widespread in Europe. *Tinea*, particularly non-bullous *tinea*, has to be considered as a possible differential diagnosis of *impetigo contagiosa* [9].

The following study describes an outbreak of *T. violaceum* in a German kindergarten that was first reported as impetigo contagiosa. Additionally, studies on the epidemiology of *T. violaceum* in Europe were analyzed.

## Methods

### Case investigations and infection control measures

Case investigations and infection control measures were carried out according to the German Infection Protection Act (IfSG), sections 16, 25, 28, and 34. Disinfection and cleaning procedures corresponded to the guideline “*Tinea capitis*” of the German Society of Hospital Hygiene [10]. As a surface disinfectant, Terralin^®^ liquid (25 g ethanol [94%]/100 g, 35 g propan-1-ol/100 g) was applied. Although Terralin^®^ liquid is not declared as fungicidal, its spectrum of activity includes dermatophytes such as *M. audouinii* and *T. gypseum* as well as molds within the declared exposure time of 5 minutes [11].

### Microbiological diagnostics

Mycological diagnostics included direct microscopy of skin scrapings taken from the actively growing edge of the suspicious skin areas and placed in 7.5% potassium hydroxide solution. Sabouraud’s dextrose agar was used for fungal cultures in Petri dishes. In the second reported case, a polymerase chain reaction (PCR) test for the *Trichophyton rubrum* complex and further PCR diagnostics were carried out to identify the pathogen in a special microbiological laboratory. The latter comprised PCR testing after additional sequencing of the entire internal transcribed spacer area of the ribosomal deoxyribonucleic acid and the translation elongation factor 1-alpha with a subsequent database comparison of the sequencing results.

### Literature search and analysis of data

A literature search in Medline was conducted on 8 December 2020 using the following search terms: “Outbreak”, “*Trichophyton violaceum*”, and “Europe”. Results were limited to the period between 1 January 2000 and 8 December 2020. Forty-six results were found and evaluated. Only original papers containing epidemiological data were included and analyzed (37 studies). For statistical analysis, the Wilcoxon rank-sum test was used to test whether the median value differed between groups (level of significance p<0.05). Statistics were done using Microsoft Excel 2016 and a web-based calculator for the Wilcoxon rank-sum test [12]. Further relevant literature was included selectively for discussion.

## Results

### Outbreak report

On 2 January 2019, the responsible public health department was notified by a municipal childcare facility about 4 educators with symptoms of *impetigo contagiosa*. In the respective kindergarten, about 70 children – mainly from families seeking asylum – were cared for by 18 educators. First, the health department gave advice on hygiene measures such as disinfecting and cleaning procedures. Second, all affected persons were recommended to consult a dermatologist. A ban on visiting the facility for up to 24 hours after the start of effective antibiotic therapy or until healing of all affected skin areas was issued for the cases [13]. Further cases were reported in the week after the detection of the outbreak: 3 employees, the husband of an infected employee, and the husband of a previously unaffected employee. At this stage, dermatologists suspected dermatomycosis possibly transmitted by a pet. At that time, 7 educators and 2 household contact persons were affected; none of the affected persons had previously travelled abroad. 

Because initially only employees of the kindergarten and their household members but none of the children were affected, the investigations first concentrated on the pets of the employees. Because a farm was also identified as a possible source of infection, the entire animal population (4 dogs, 3 cats, and 25 chickens) was examined by a veterinarian, which did not result in any pathological findings.

Because of the clinical diagnosis of *tinea corporis*, topical therapy was started, consisting of a cyclopyroxolamine cream applied to the affected skin areas of the patients. The efflorescence of the *tinea* was found in the area of the face and neck and, in 1 patient, on the arm; the capillitium was not affected in any of the initial cases. *Microsporum audouinii* was also considered to be a potential pathogen, because about 90% of the children in the facility were from migrant families (including 13 children from Africa). The facility was closed for two weeks [10], during which the entire premises were cleaned and disinfected in 300 working hours on 5 consecutive days. A total of 40 L of the surface disinfectant Terralin^®^ liquid was applied. All objects and surfaces were disinfected (games, books, materials for handicrafts and painting, balls from the ball pit, pictures, and pieces of furniture, beds, and decorations). The public health department ordered every employee and child of the kindergarten to be examined by a dermatologist and to present a medical certificate before re-admission.

**Figure 1:**
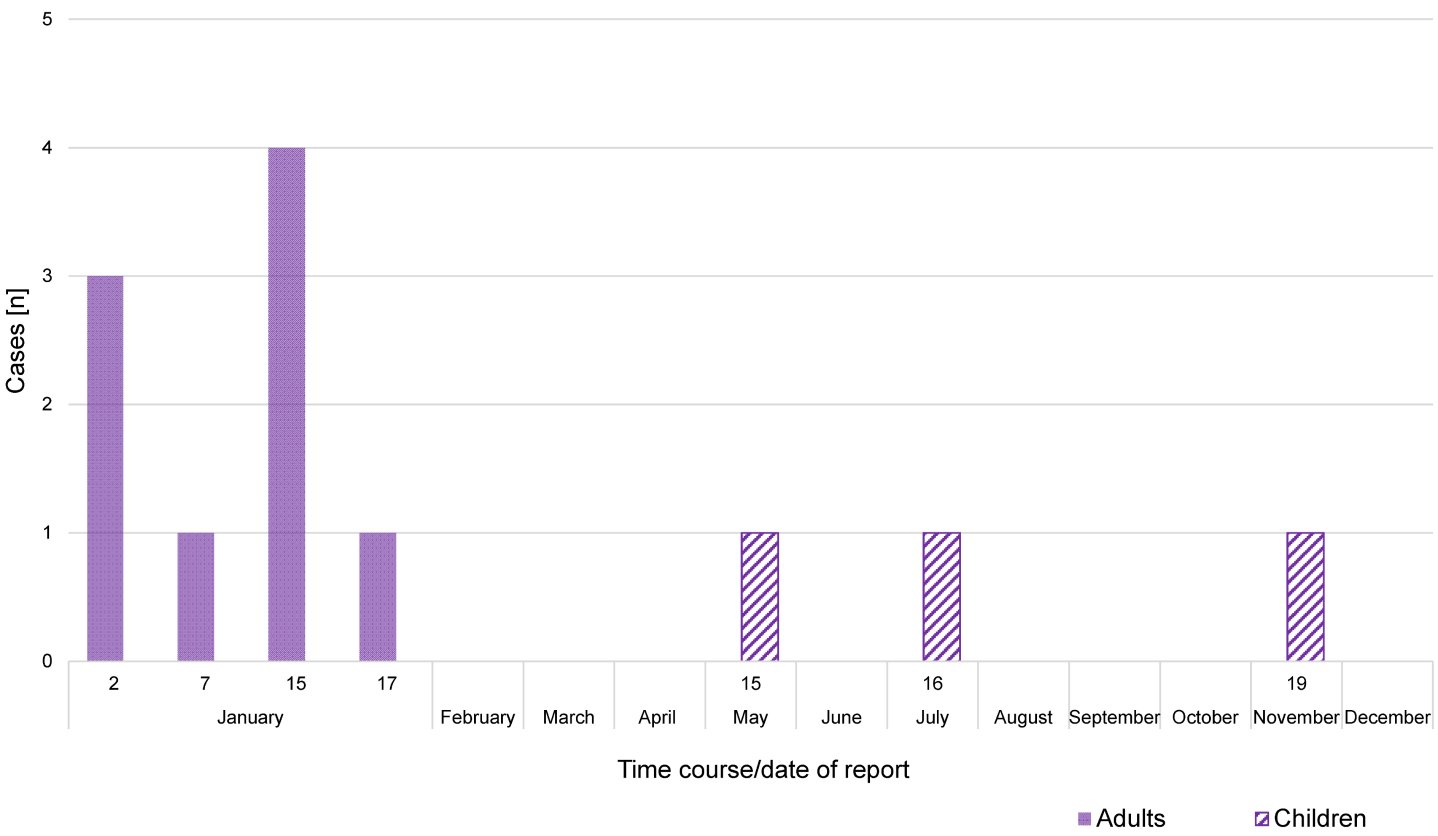
Epidemic curve of an outbreak of tinea in a German kindergarten in 2019

Overall, 9 cases of tinea had occurred by the end of January 2019 (7 educators and 2 household members; see Figure 1 (Fig. 1)). No further cases, in particular no children, had been reported by that time.

The *Trichophyton rubrum* complex was detected in 1 employee in both the PCR test and the fungal cultures. Further diagnostics were initiated because the culture showed an unusual white color. A rare, white variant of *Trichophyton violaceum*, a species of the *Trichophyton rubrum* complex, was detected in the PCR test. Despite multiple attempts, fungal cultures of the other affected persons did not yield any results, probably because of the antimycotic treatment initiated previously.

Four months after the diagnosis of the last case, a child at the kindergarten (of Nigerian descent) was diagnosed with tinea capitis, and *Trichophyton rubrum* could be cultured. No further measures were initiated, since no connection with the outbreak in the kindergarten was established because of impaired communication when the patient was examined by the dermatologist. Systemic therapy was administered without any complications until a negative culture was obtained.

**Figure 2:**
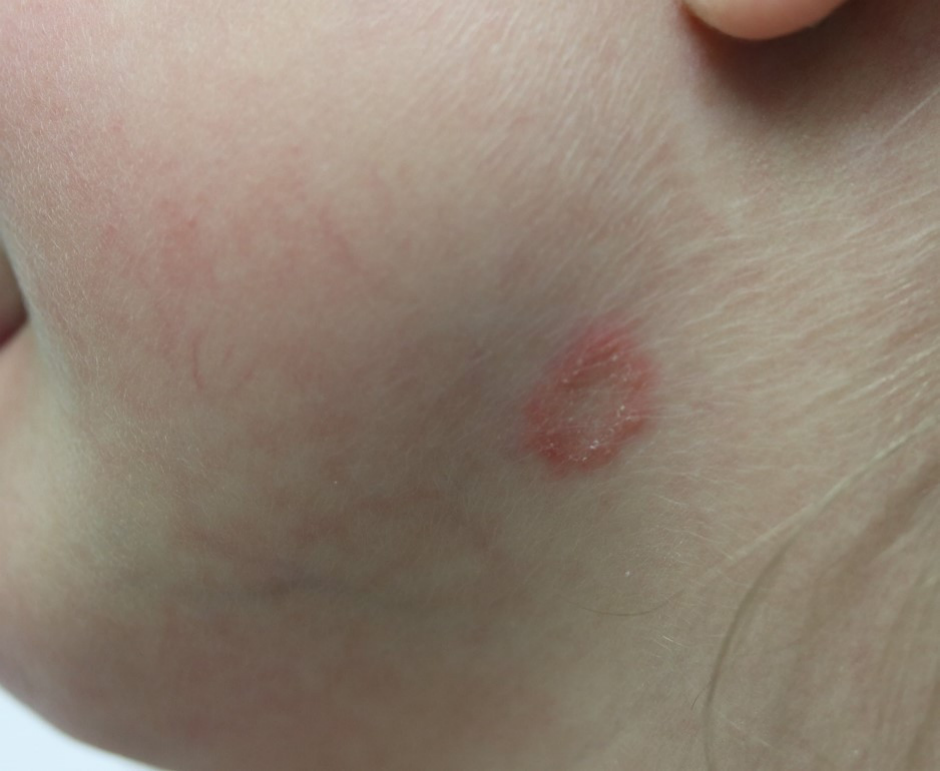
Manifestation of *Trichophyton violaceum* on the neck (photo: S. Karrer)

Another case became known in July 2019. Because of the diagnosis of *tinea corporis*, the patient was examined by a dermatologist who initiated calculated topical therapy with cyclopyroxolamine cream. In November 2019, the last known case was a child from the kindergarten who also showed the white variant of *T. violaceum*. Clinically, the patient showed discreet scaling on the left side of the neck (Figure 2 (Fig. 2)). 

### Analysis of studies from Europe

The 37 studies analyzed [14], [15], [16], [17], [18], [19], [20], [21], [22], [23], [24], [25], [26], [27], [28], [29], [30], [31], [32], [33], [34], [35], ,[36] [37], [38], [39], [40], [41], [42], [43], [44], [45], [46], [47], [48], [49], [50] had been conducted in Italy [16], [19], [20], [21], [22], [40], [46], Spain [24], [27], [29], [33], [43], [47], Greece [17], [23], [25], [31], [37], [41], Switzerland [14], [30], [45], the UK [32], [44], Sweden [34], [42], Croatia [35], [48], Slovakia [15], the Republic of Macedonia [26], the Republic of Ireland [28], Bosnia and Herzegovina [36], Belgium [38], Malta [39], France [49], and Slovenia [50]. 26 of 37 publications (70%) were from southern and southeastern European countries. Most studies were retrospective; 3 studies were prospective or cross sectional [20], [33], [47], and 1 was a case report [16]. 

Seventeen studies focused on the epidemiology of dermatophytoses and the prevalence of *T. violaceum* in dermatophytic and dermatomycotic infections [15], [17], [18], [23], [26], [27], [29], [35], [39], [41], [42], [44], [45], [46], [47], [48], [50]. Thirteen studies reported mainly on tinea capitis [20], [24], [28], [30], [31], [32], [33], [34], [36], [37], [38], [43], [49]. Five studies only included children [20], [28], [33], [34], [38]. Six studies only reported on infections with *T. violaceum* [14], [16], [19], [21], [25], [40] (Table 1 (Tab. 1)), and 1 study solely focused on *tinea manuum* [20]. 

Three studies on *T. violaceum* in different age groups provided detailed data on the age of patients with *T. violaceum* [19], [21], [31]. The mean age of the overall reported 78 patients was 20.1 years (median age 8 years, IQR: 4.8–38.0). Seven studies presented detailed data on the nationality of the patients [20], [21], [25], [31], [32], [38], [40]: 145/277 (52.3%) patients with *T. violaceum* dermatophytosis were of African descent (most frequently from Ethiopia [29%], Egypt [9.7%], and Somalia [6.9%]), 123 (44.4%) from Europe, and 9 (3.2%) from Asia. 

Sample numbers of dermatophytosis and prevalence of *T. violaceum* were available or could be calculated from 14 studies [17], [23], [26], [27], [29], [35], [39], [41], [42], [44], [45], [47], [48], [50]. Both sample size and prevalence showed considerable variance (Table 2 (Tab. 2)). The sample size ranged from 238 to 15,333 samples. The median prevalence of all studies was 1.7% (IQR: 0.7% to 4.8%). No trend over time concerning the prevalence of *T. violaceum* was detected between 2002 and 2016.

**Figure 3:**
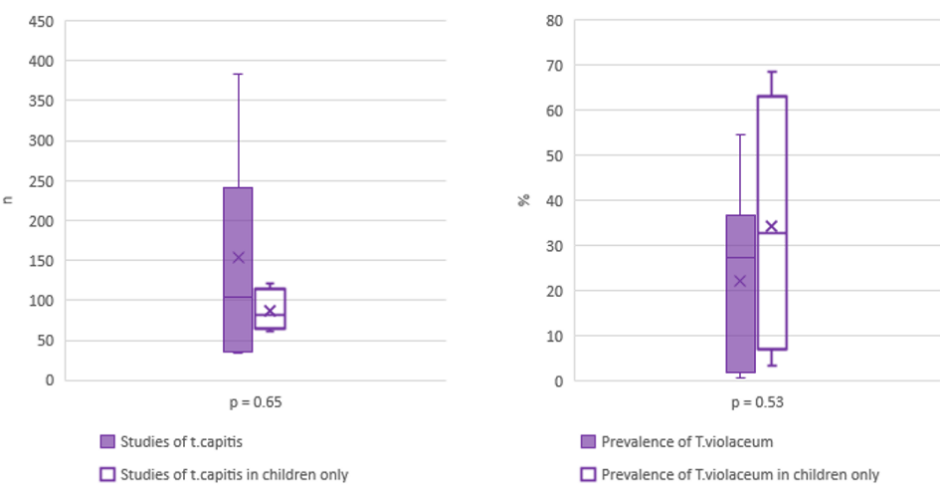
Reported case numbers of *Tinea capitis* (left) and prevalence of *Trichophyton violaceum* (right) reported in studies focusing on *Tinea capitis* in the general population [24], [30, [32], [36], [37], [43], [49] and in children only [20], [28], [34], [38].

Eleven studies reported cases of *T. capitis* [20], [24], [28], [30], [32], [34], [36], [37], [38], [43], [49]. The median prevalence of *T. violaceum* was 27.3% (IQR: 3.2% to 47.2%), and the number of patients diagnosed with *T. capitis* ranged from 33 to 383. Taking only the studies into account that reported on *T. capitis* in children [20], [28], [34], [38], the case numbers ranged from 70 to 120, with a median prevalence of 32.6% for *T. violaceum* (IQR: 6.9% to 63.2%). The comparison of case numbers and prevalence of *T. violaceum* between studies focusing on *T. capitis* in general [22], [28], [30], [34], [35], [41], [47] and those on *T. capitis* in children only [20], [28], [34], [38] did not yield statistically significant differences (Figure 3 (Fig. 3)); the median prevalence of *T. capitis* in general was 27.3% and 32.6% in children only. 

In one study [31] of 1360 patients with *T. capitis*, 34 household members of 15 patients were screened for asymptomatic carriage; 97% were asymptomatic carriers, and 59% showed infection with *T. violaceum*.

## Discussion

In the described outbreak, fungal skin disease – initially diagnosed as *Impetigo contagiosa* – arose in a kindergarten; later on, *Trichophyton rubrum* complex was detected in 2 patients. Although this pathogen was not detected in the other cases, an outbreak had to be assumed because of the epidemiological connection. Ideally, cultures should be started before the initiation of a calculated therapy, but treatment must not be delayed because of potential complications or spread of the infection. For epidemiological reasons, the underlying pathogen should be identified (or genotyped) in all affected persons.

In the present case, the source of infection and the chain of transmission remained unclear. Examination by a dermatologist and the collection of mycological samples and cultures for all household members and persons with close contact to the children (approx. 70 children) initially appeared disproportionate, as it was a delayed outbreak. Furthermore, any possible positive results of the mycological cultures would have taken up to 8 weeks and, in the case of asymptomatic carriers, would not have had any therapeutic consequences, because positive results are not an indication for systemic therapy (in children, systemic therapy constitutes off-label use, and “complete” treatment without any visible manifestation may be difficult to achieve with topical application only). In the case of an outbreak, some authors have recommended extensive screening of all contact persons, for instance, by using a procedure termed hairbrush method, and the treatment of asymptomatic carriers [6], [51], [52]. However, the question arises as to how sensitive this method is if other body parts apart from the scalp are affected (macroscopically not at all or only barely visible). Moreover, neither the hairbrush method nor detection by means of a PCR test are currently standard procedures in mycological diagnostics. Because of the plasticity of fungi, different types of dermatophytes may look the same, whereas the same dermatophytes may look different, which makes reliable identification of the pathogen in cultures difficult [53]. PCR tests have not yet been adequately established for certain pathogens, but may at least partially solve this problem in the future [52], [53], [54]. Furthermore, *T. violaceum* is not a highly virulent pathogen; thus, it would have been disproportionate to close a facility until the results of the cultures of all contact persons were available. Because of the low dynamics of the described outbreak, the authors favored active surveillance and regular contact with the facility to detect any new cases or a new outbreak at an early stage.

Studies on the epidemiology of *T. violaceum* from Europe report a different prevalence of *T. violaceum*, depending on the assessed population or the number of samples examined. Most studies were conducted in southern or southeastern European countries. A higher prevalence of *T. violaceum* is seen in people of African descent [20], [21], [25], [31], [32], [38], [40], indicating an obvious association with migration. Infections with *T. violaceum* mainly appear clinically as *T. capitis* and are most prevalent in children [17], [18], [19], [20], [21], [22], [23], [24], [25], [26], [27], [28], [29], [30], [31], [32], [33], [34], [35], [36], [37], [38], [39], [40], [41], [42], [43], [44], [45], [46], [47], [48], [49], [50], although our analysis did not yield any statistically significant difference in prevalence between the studies on *T. capitis* in general and those on children only. Asymptomatic carriage seems to be important with regard to the spread of *T. violaceum*, but this phenomenon has been seldom addressed in clinical studies [31]. Overall, due to the heterogeneity of the populations considered in the available studies, there is no clear trend with regard to the prevalence of *T. violaceum* over time. Limitations of this study are its secondary evaluation and the highly selective underlying primary data. 

## Conclusions

Global mobility is associated with the (re)occurrence of “unusual” infectious agents. PCR diagnostics will increasingly become relevant for mycotic diseases in the near future. In case of dermatophytic outbreaks, extensive screening and treatment of asymptomatic carriers must be considered after risk-benefit assessment, depending on the dynamics of the individual outbreak. Data on the epidemiology of *T. violaceum* in Europe are heterogeneous, scarce and limited by selection bias.

## Notes

### Conflict of interest

The authors declare that there is no conflict of interest.

### Funding

No funding was received. 

### Acknowledgement 

We thank Professor Pietro Nenoff for conducting the specific PCR diagnostics and Monika Schöll for the linguistic revision of the manuscript.

## Figures and Tables

**Table 1:**
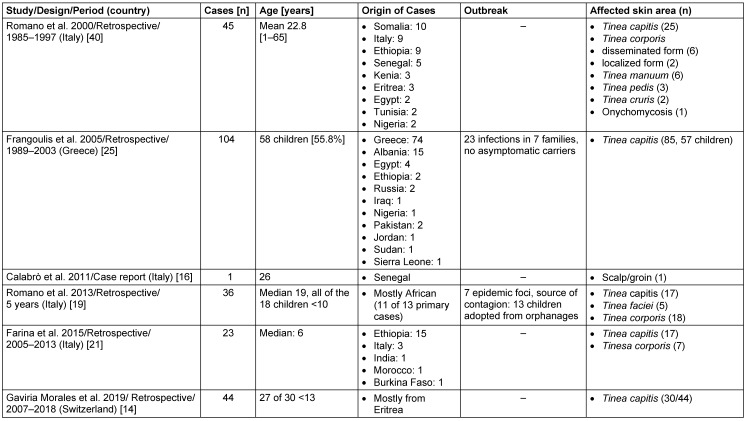
European studies on dermal infection with *Trichophyton violaceum* only (6 of 37, ranked by year of publication)

**Table 2:**
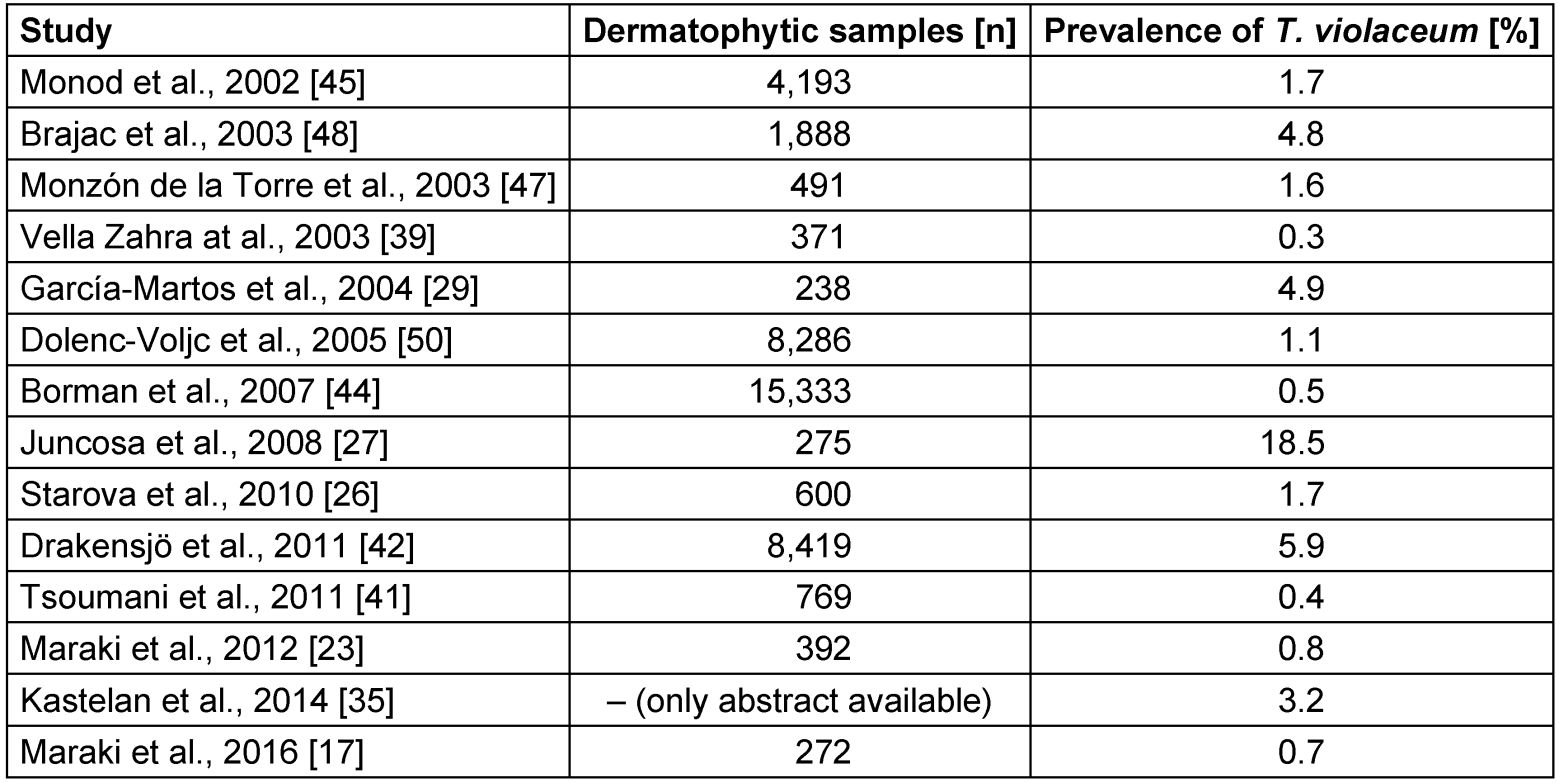
Dermatophytic samples examined and prevalence of *Trichophyton violaceum* in 14 European studies (ranked by year of publication)
